# Double-Blind, Placebo-Controlled Trial of Cyproterone Acetate to Prevent Flare-Up Effect on Dogs Implanted With Deslorelin

**DOI:** 10.3389/fvets.2021.714154

**Published:** 2021-09-29

**Authors:** Sylvia Masson, Tiphaine Medam, Elsa Raibon, Christelle Fontaine, Xavier Levy

**Affiliations:** ^1^Clinique de la Tivolliere, Voreppe, France; ^2^Independent Researcher, Jouques, France; ^3^Virbac, Carros, France; ^4^Centre de Reproduction des Carnivores du Sud-Ouest (CRECS), L'isle Jourdain, France

**Keywords:** deslorelin, flare-up, cyproterone acetate, urine marking, aggressivity, mounting, dog medical castration

## Abstract

Deslorelin slow-released implants are registered in Europe for the reversible suppression of fertility in male dogs. After administration, a time-limited increase in sex hormones concentration and related behavioral problems may be observed. The aim of this work was to assess whether cyproterone acetate, a synthetic progestogen, can prevent this flare-up effect. Eighteen privately-owned entire male dogs were enrolled in this double-blind, placebo-controlled, randomized clinical trial. All subjects received a 4.7 mg deslorelin implant by SC route and 1–3 capsules containing either cyproterone acetate 2 mg/kg (*N* = 9) or a placebo (*N* = 9), by oral route BID for 14 days, depending on the dog's weight. The dogs were followed for 28 days. An increase in the blood testosterone concentration was observed in respectively 9/9 and 7/9 dogs of the control and cyproterone groups (*p* = 0.47). However, a worsening of the sex hormone related problems (i.e., urinary marking, mounting, aggressiveness toward other dogs and/or escape) was only observed in the placebo group, in 56 or 66% of the dogs as measured by respectively the veterinarian and the owners. Our study suggests that cyproterone acetate is effective and safe to supress the deslorelin induced behavioral flare-up effect, but not the rise in testosterone.

## Introduction

Gonadotropin-releasing hormone (GnRH) agonists slow-release implants such as deslorelin or azaglynafarelin have become more and more popular in veterinary medicine since their commercialization in the European Union in 2008 (1, 2). In companion animals, the deslorelin implant (Suprelorin®, Virbac, Carros, France) is the most commonly used. Chemical castration is an ethically interesting alternative to surgical castration and can improve the dog immediate welfare because the implant is reported to induce the same positive effect as surgical castration with regard to aggression, fear and play behavior (3) while avoiding a general anesthesia and pain from the surgery (4–6). Moreover, in many cases, the veterinarian does not know if the orchiectomy will induce the change expected by the owners if the request is of behavioral nature. Thus, a deslorelin implant can be a good option when neutering is considered to solve behavioral problems because its effects are reversible.

GnRH is a decapeptide synthesized by the hypothalamus and has a very stable sequence in all mammals (7). Analogs of this decapeptide provides both GnRH agonists and antagonists. Suprelorin® has two major effects: a stimulation or the GnRH receptors, followed by a desensitization of the same receptors to GnRH. The stimulation of the GnRH receptors leads to an increase in FSH and LH, and consequently to an increased release of sex hormones (i.e., testosterone in male or estrogenes in females) (8). The elevation of the gonadotropins and sex hormones during the stimulation phase is called the flare-up (FU) effect. In general, an increase in plasma levels of testosterone is observed as early as 20 min after the deslorelin implant has been injected and rapidly declines to basal values to reach undetectable concentrations ~12 days (range 6–25) after implantation (9). Conversely, the desensitization of the GnRH receptors to GnRH lead to a long term fully reversible downregulation of testicular endocrine function in male dogs (4, 5).

Consequently, GnRH agonists may either stimulate estrus or effectively sterilize the dog, depending on the expected effect, i.e., FU effect or GnRH desensitization. This double mechanism of action explains why suprelorin has been extensively used over the years, in a wide range of clinical applications and in many species: male sterilization in dogs (4, 8–12), œstrus induction in female dogs (13–16), œstrus inhibition in female cats (17–20), contraception and prevention of adrenal disease in male ferrets (21–23) as well as females (24), estrus supression in ferrets (25), delaying puberty in male dogs (26, 27) and cats (28, 29), management of benign prostatic hyperplasia (9, 30), and treatment of sex hormone-related behavioral problems (31–33) in dogs.

However, when used in male dogs for sterilization purpose or to assess the potential efficacy of neutering on behavioral problems, the FU effect constitutes a potential annoying side effect (31). Indeed, the clinical signs that are targeted may actually worsen for up to 15 days after implantation, and FU could also lead to apparition of new behavioral problems. Depending on the reported issue, this side effect can be considered as slightly annoying for owners (e.g., urine marking, mounting) or become dangerous for the dog itself (e.g., runaway) or other dogs or people (e.g., aggressivity). Moreover, based on informal feedback from on the field (unpublished data from the laboratory), veterinarians often express concerns that their responsibility may be engaged in case of worsening behavior following implantation. Hence, finding a molecule that could be able to block the FU effect and/or its consequences could be of remarkable interest. Progestins have been reported as partially successful in preventing induced estrus after deslorelin implantation in female dogs (5, 14, 34). Looking for potential molecules, cyproterone acetate appeared to be a good candidate because of its very particular mechanism of action that adds a behavioral effect to the classical progestin effect coming from testosterone receptors blockade (35).

Cyproterone acetate is a synthetic progestogen with a wide range of mechanisms of action (36). Firstly, it acts via a double mechanism: on one hand it blocks androgens peripherical action via a competitive inhibition to the testosterone cytosolic receptor, and on the other hand it blocks GnRH secretion leading to a secondary blockage of FSH, LH and testosterone (37). This double mechanism could result in testosterone circulating levels that are lower than those observed with chemical castration (38). Moreover, it has been established that cyproterone acetate interacts with the GABA receptor subtype A, which is known to have anticonvulsant and anxiolytic properties (36, 37). This progestin may also reduce levels of 5-hydroxy indole acetic acid and homo vanillic acid, metabolites of serotonin and dopamine, resulting in an increase in the availability of these monoamines in the central nervous system (37). Another potential mode of action is that cyproterone may modulate the enzyme 5-alpha reductase, thereby altering the brain concentration of allopregnanolone, a neurosteroid synthesized both in neurons and glia that has anxiolytic properties at high doses (39). Finally, cyproterone acetate was shown to bind to opiate receptors in mice with a potential role in the endorphin system (37). In humans, cyproterone acetate has been used in the treatment of prostate cancer and is now used for androgen-dependent indications in women and precocious puberty in boys. It has also been used to control unresponsive aggression in aggressive dementia, and as sex offender treatment (40–42). Cyproterone acetate has a weak glucocorticoid activity, which renders its use safer than any other progestins. However, several authors reported mild, mostly reversible, adverse effects such as weight gain, loss of libido and gynecomastia, which are due to the decreased testosterone concentrations in serum (41). In addition, hepatotoxicity has been described and can lead to fatal acute liver failure induced by cyproterone acetate (43). However, such toxicity was always associated with an increase of alanine aminotransferase (ALT) and aspartate aminotransferase (AST) activities, ranging from 6 to 59 times the upper normal levels (43). For this reason, monitoring the liver function following any cyproterone acetate treatment is mandatory. Thus, we hypothesized that cyproterone acetate could efficiently block the FU effect in dogs after a deslorelin implantation.

## Materials and Methods

This study was approved by an independent yet internal Ethical Review Committee (ERC# 2OL9O9-1), stating that the protocol complied with the European Directive # 2010-63-EU, the application of the 3Rs principles and the internal Code of Animal Care, as the study occurred in privately owned veterinarian clinics.

### Subjects' Recruitment

The subjects of this study were adult, healthy, entire, domestic male dogs (*Canis familaris*) whose owners requested a chemical castration because of urinary marking, aggressivity toward other dogs, escaping and/or mounting behaviors. Such behavioral issues could have environmental, psychiatric (i.e., neuropathological) or hormonal origins. They could have existed for years without being addressed by the owners or just recently have been discovered by the owners after the dog's adoption. However, these variables were not considered in the inclusion or exclusion criteria since they were irrelevant for the purpose of our study (namely, investigating the effect of cyproterone acetate on the FU effect).

In other words, dogs were included in our study if and only if they were entire (neither cryptorchid nor monorchid) males, were over 6 months of age (i.e., concerned by hormonal puberty) and exhibited one or several of the aforementioned behavioral symptoms (urinary marking, aggressivity toward other dogs, escaping and/or mounting behaviors). Aggressivity toward humans, other medication than vaccines and deworming treatments for the last 15 days or previous deslorelin implantation were considered as exclusion criteria.

Data were collected on a total of 18 dogs which had been recruited during routine visits or behavioral consultations by the clinical investigators or their collaborators. All the owners of dogs meeting the inclusion criteria were explained the trial and asked to recontact the clinical investigators if they wanted to participate. Interested owners were then given all the necessary instructions for which they gave their informed consent.

### General Procedure

The study was designed as a double-blind, placebo-controlled clinical trial: the 18 subjects were alternatively assigned to either the cyproterone acetate group (*N* = 9) or the Placebo group (*N* = 9), following an order which was counterbalanced across the clinical investigators.

The first day of the study (D0), the clinical investigators implanted all the subjects with a deslorelin implant and estimated the frequency and severity of their symptoms, based on the owners' reports, in order to establish a point of comparison for the follow-up visits (described below).

Twice a day and for 14 days, all subjects were orally administered by their owners one to three capsules, depending on the dog's weight (see Table 1), of either cyproterone acetate (for the cyproterone acetate group) or placebo (for the placebo group), without the dogs' owners nor the clinical investigators being aware of the treatment allocation.

**Table 1:** Posology of the cyproterone acetate in the study.

**Dog's weight (kg)**	**Posology (morning and evening)**
5–9	One 10 mg capsule
10–14	One 20 mg capsule
15–19	One 30 mg capsule
20–24	Two 20 mg capsules
25–29	One 30 mg capsule and one 20 mg capsule
30–39	Two 30 mg capsules
40–49	Two 30 mg capsules and one 20 mg capsule
50–59	Three 30 mg capsules

To ensure blinding, the clinical investigator asked a pharmacist (Delpech Marseille) to compound cyproterone acetate capsules from commercially available pills (ANDROCUR 50 mg) and placebo capsules filled with cellulose powder, into capsules looking alike and labeled A or B. The pills were compounded using the human formulation of cyproterone acetate, since this drug is not labeled for veterinary medicine in France. Three dosages (10, 20, and 30 mg) were available for both A and B pills. Dogs were alternatively allocated to treatments A and B according to the order of recruitment in each clinic participating in the study. Two clinics started with case B first, one started with case A first.

Besides the first one (D0), the study comprised seven test phases or visits at the clinical investigators' clinics which respectively took place 1 day (D1), 3 days (D3), 5 days (D5), 7 days (D7), 10 days (D10), 14 days (D14) and 28 days (D28) after D0. Each visit (with the exception of the last one) consisted in (i) a veterinarian-based evaluation of the dog's behaviors (scored −10 to +10) compared to this dog's behaviors (scored 0) at D0, (ii) the collection of two 0.5 mL blood serum samples to measure the dog's testosterone level and (iii) the recording of the potential adverse effects of the treatment. This behavioral score was based on the compared frequency and severity of the five recorded symptoms considering that the problem was completely solved for a score of 10, not changed at 0 and deeply worsened at −10. All analyses of the dogs' testosterone levels were conducted at once, after the 18 dogs included in the study had finished the protocol, using VIDAS® Fertilité (Biomerieux®). In addition, a full blood sample to run blood count and biochemistry were collected on D0 and D10, to check for possible hepatic effects on renal and hepatic functions. Each investigator used his own laboratory material (i.e., Idexx Catalyst® and Idexx Lasercyte® for two clinics, Idexx VetTest®, and Idexx Lasercyte® for the last one) to run the blood count and biochemistry, so there were three different machines used for the whole study, but only one machine used for each dog. The dog's weight was recorded on D0, D10, and D28 to check for possible weight gain effects. Finally, owners were asked at D28 their level of satisfaction concerning the implant's efficiency on the behavior of their dog and they were asked if they saw a worsening in the behavior in the first 10 days (i.e., if they observed a FU effect).

Considering the specificity of the protocol with a number of visits and blood sampling, the three investigators were selected because they had a diploma in behavior (French diploma of behavioral medicine) and were trained to positive training practices and gentle handling which would limit the possible negative impact of the repeated sampling on the participating dogs.

### Investigated Items and Statistical Analyses

Preliminary analyses aimed to ensure not only that the subjects' characteristics (i.e., breed, age, weight, testosterone level, and symptoms) at D0 were not significantly different in the two treatment groups (i.e., placebo and cyproterone acetate), but also that the physiological and behavioral manifestations of the testosterone FU effect were indeed correlated. Primary analyses aimed to evaluate the effect of the administered treatment (cyproterone acetate or placebo) on the subjects' testosterone levels and behavioral symptoms. The former was assessed by the statistical comparison of the cyproterone acetate group subjects' and placebo group subjects' percentage change in testosterone level compared to D0. The latter was first and foremost assessed by the statistical comparison of the cyproterone acetate group subjects' and placebo group subjects' average and symptom-specific behavioral scores, ranging from −10 (i.e., extreme deterioration) to +10 (i.e., extreme improvement), compared to D0 (i.e., 0). Finally, secondary analyses aimed to determine whether the subjects' experienced adverse effects and weight gain (if any) and whether the owners' satisfaction scores concerning the overall effect of the implantation were significantly different in the two treatment groups (i.e., placebo and cyproterone acetate). All statistical analyses were conducted using R statistical software (https://www.rproject.org) and adopting a significance threshold of 0.05. All data were analyzed with 2-tailed tests. Statistical comparisons between groups were made by use of Student *t*-tests when we could confirm normal distributions with the Shapiro-Wilk test and *F*-test for equality of variances, and by use of Mann-Whitney tests otherwise. Statistical comparisons within groups were made by use of Student *t*-tests after confirming normal distributions with the Shapiro-Wilk test and *F*-test for equality of variances. In addition, Freeman-Halton extensions of the Fisher exact probability test were performed to examine the relation between some qualitative variables.

## Results

### Preliminary Analyses

#### Demographics

A first set of analyses aimed to ensure that the subjects' characteristics (i.e., breed, age, weight, testosterone level, and symptoms) at D0 were not significantly different in the two treatment groups (i.e., Placebo and cyproterone acetate) and therefore could not account for the effects of these treatments, if any. Because none of the included dogs were of the same breed in our study (see Table 2 below), this factor could not be checked and was dropped from all the analyses.

**Table 2:** Numbers of subjects by breed in the two treatment groups.

	**Group**	
**Breed**	**Placebo**	**Cyproterone acetate**
American Staffordshire Terrier	0	1
Australian Shepherd	0	1
Beagle Mix	0	1
Bernese Mountain Dog	0	1
Coton de Tuléar	0	1
French Bulldog	0	1
German Shepherd	0	1
Staffordshire Bull Terrier	0	1
Yorkshire Terrier	0	1
Beauceron	1	0
Chihuahua Mix	1	0
English Setter	1	0
Jack Russel Terrier	1	0
Miniature Spitz	1	0
Rottweiler	1	0
Setter X Australian Shepherd	1	0
Shih-tzu	1	0
Weimaraner	1	0

The subjects' ages (in months) at D0 in the placebo group (*M* = 34.2; *SD* = 26.7) and the cyproterone acetate group (*M* = 54.6; *SD* = 35.9) were compared using a parametric test for independent samples since they had normal distributions (Shapiro-Wilk normality tests: *W* = 0.87 and *p* = 0.12 for the placebo group; *W* = 0.92 and *p* = 0.41 for the cyproterone Acetate group) and equal variances [*F*-test: *F*_(8,8)_ = 1.81; *p* = 0.42]. This two-samples *t*-test shows that the subjects' ages at D0 were not significantly different between the two treatment groups [*t*_(16)_ = 1.36; *p* = 0.19].

The subjects' weights (in kilograms) at D0 in the placebo group (*M* = 21.7; *SD* = 16.0) and the cyproterone acetate group (*M* = 23.4; *SD* = 13.7) were compared using a parametric test for independent samples since they had normal distributions (Shapiro-Wilk normality tests: *W* = 0.90 and *p* = 0.24 for the placebo group; *W* = 0.96 and *p* = 0.84 for the cyproterone acetate group) and equal variances [*F*-test: *F*_(8,8)_ = 0.73; *p* = 0.67]. This two-samples *t*-test shows that the subjects' weights at D0 were not significantly different between the two treatment groups [*t*_(16)_ = 0.25; *p* = 0.81].

The subjects' testosterone levels (in nanograms per milliliter of blood) at D0 in the placebo group (*M* = 4.6; *SD* = 3.8) and the cyproterone acetate group (*M* = 4.0; *SD* = 3.4) were compared using a parametric test for independent samples since they had normal distributions (Shapiro-Wilk normality tests: *W* = 0.94 and *p* =0.54 for the placebo group; *W* = 0.85 and *p* = 0.07 for the cyproterone acetate group) and equal variances [*F*-test: *F*_(8,8)_ = 0.77; *p* = 0.72]. This two-samples *t*-test shows that the subjects' testosterone levels at D0 were not significantly different between the two treatment groups [*t*_(16)_ = −0.30; *p* = 0.77].

The subjects' symptoms (i.e., urinary marking, mounting, aggressivity toward other dogs, and/or escape) at D0 in the placebo group and the cyproterone acetate group were analyzed using the Freeman-Halton extension of the Fisher exact probability test for a two-rows by four-columns contingency table (see Table 3 above), which shows that the proportions of each symptom did not differ by treatment groups (*p* = 0.88).

**Table 3:** Numbers of subjects by symptoms in the two treatment groups.

	**Group**	
**Symptoms**	**Placebo**	**Cyproterone acetate**
Urinary Marking	8	6
Mounting	7	7
Aggressivity toward other dogs	5	7
Runaway	3	4

#### Relation Between the Behavioral and Physiological Aspects of Testosterone Flare

The testosterone flare-up effect can manifest itself at two different levels, namely (i) in an increased testosterone level (i.e., a testosterone level higher than that of reference measured at D0) that will hereafter be called the *physiological* flare-up effect (PFU) as well as (ii) in an increased severity of behavioral symptoms (i.e., behavioral scores smaller than that of reference, 0, attributed at D0) that will hereafter be called the *behavioral* flare-up effect (BFU). The BFU is supposedly a consequence of the PFU, but this assumption had to be verified before analyzing the effect of the treatment (placebo or cyproterone acetate) on the subjects' FU effect. Thus, a second set of analyses aimed to ascertain that PFU and BFU co-occurred (in a subject and/or over time) andwere, indeed, two different aspects of one phenomenon (the FU effect).

A Freeman-Halton extension of the Fisher exact probability test for a two-rows by two-columns contingency table (see Table 4 above) was performed to examine the relation between the presence of a PFU (Yes/No) and the presence BFU (Yes/No) in the subjects. It showed that there was no significant association between these two variables (*p* = 0.53), even though all of the (6) subjects experiencing a BFU effect also presented a PFU effect. Because of this very small number of subjects experiencing both a PFU and a BFU, no analysis on the temporal patterns of these two variables (see Table 5 below) were carried out. However, it has to be noted that, when both a PFU and a BFU occurred in one given subject (*N* = 6), they never did on the same exact period: the PFU began before (50%) or with (33%) the BFU in 83% of the cases (so that only one subject experienced the BFU before the PFU) and finished before (50%) or after (50%) the BFU in 100% of the cases.

**Table 4:** Numbers of subjects experiencing or not PFU and/or BFU.

	**BFU**	
**PFU**	**No**	**Yes**
*No*	2	0
*Yes*	10	6

**Table 5:** Start day, end day, and duration of PFU and BFU occurring in the same subject.

	**Start day**		**End day**		**Duration**	
**Subject**	**PFU**	**BFU**	**PFU**	**BFU**	**PFU**	**BFU**
3	1	3	7	3	7	1
5	3	5	3	10	1	6
7	1	1	10	3	10	3
9	3	1	3	10	1	10
11	1	1	3	5	3	5
15	1	3	7	3	7	1
M	1.7	2.3	5.5	5.7	4.8	4.3
SD	1.0	1.6	2.9	3.4	3.7	3.4

Overall, these results indicate that, even though the behavioral manifestation of the testosterone FU (i.e., the BFU) did not happen without and tended to begin with or after (but can continue beyond) the physiological manifestation of the testosterone FU (i.e., PFU), these two aspects of the testosterone FU effect had to be considered independently in subsequent analyses. Moreover, because the temporal patterns of both the PFU and the BFU varied greatly from one subject to another, subsequent comparisons of these patterns between the two treatment groups had to rely on individual FU start day, end day, and duration rather than on treatment days.

### Secondary Analyses

#### Effects of The Treatment on the PFU

A Freeman-Halton extension of the Fisher exact probability test for a two-rows by two-columns contingency table (see Table 6 below) was performed to examine the relation between the subjects' experience of a PFU (Yes/No) and treatment group (placebo/cyproterone acetate) and showed that there was no significant association between these two variables (*p* = 0.47), even though 100% of the subjects from the placebo group against only 78% of the subjects from the cyproterone acetate group experienced a PFU.

**Table 6:** Numbers of subjects experiencing or not PFU in the two treatment groups.

	**Treatment group**	
**PFU**	**Placebo**	**Cyproterone acetate**
No	0	2
Yes	9	7

The subjects' *start days* of PFU in the placebo group (*M* = 1.7; *SD* = 1.0) and cyproterone acetate group (*M* = 1.0; *SD* = 0) were compared using a non-parametric test for independent samples, since Shapiro-Wilk normality tests showed that the data were not normally distributed in the placebo group (*W* = 0.62; *p* < 0.001) and could not be carried on in the cyproterone acetate group (all values being equals). This Mann-Whitney test shows that the subjects' start days of PFU were not significantly different between the two treatment groups [*U*_(7,9)_ = 21; approximate *p* = 0.12].

The subjects' *end days* of PFU in the placebo group (*M* = 5.9; *SD* = 2.8) and cyproterone acetate group (*M* = 4.9; *SD* = 2.7) were compared using a non-parametric test for independent samples, since (as shown by Shapiro-Wilk normality tests) the data were normally distributed in the placebo (*W* = 0.86; *p* = 0.11) but not the cyproterone acetate (*W* = 0.76; *p* = 0.02) group. This Mann-Whitney test shows that the subjects' end days of PFU were not significantly different between the two treatment groups [*U*_(7,9)_ = 24; approximate *p* = 0.43].

The subjects' *duration* of PFU in the placebo group (*M* = 4.2; *SD* = 3.5) and cyproterone acetate group (*M* = 3.9; *SD* = 2.7) were compared using a non-parametric test for independent samples, since (as shown by Shapiro-Wilk normality tests) the data were normally distributed in the placebo (*W* = 0.90; *p* = 0.27) but not the cyproterone acetate (*W* = 0.76; *p* = 0.02) group. This Mann-Whitney test shows that the subjects' duration of PFU were not significantly different between the two treatment groups [*U*_(7,9)_ = 30.5; approximate *p* = 0.96].

The subjects' percentage increase in testosterone levels *on the start days* of the subjects' PFU in the placebo group (*M* = 627.2; *SD* = 1678.7) and the cyproterone acetate group (*M* = 170.6; *SD* = 171.5) were compared using a non-parametric test for independent samples, since Shapiro-Wilk normality tests showed that the data were normally distributed in the cyproterone acetate group (*W* = 0.86; *p* = 0.17) but that were not in the placebo group (*W* = 0.42; *p* < 0.001). This Mann-Whitney test shows that the percentage increase in testosterone levels on the first day of the subjects' PFU were not significantly different between the two treatment groups [*U*_(7,9)_ = 40; *p* = 0.41].

**Figure 1:**
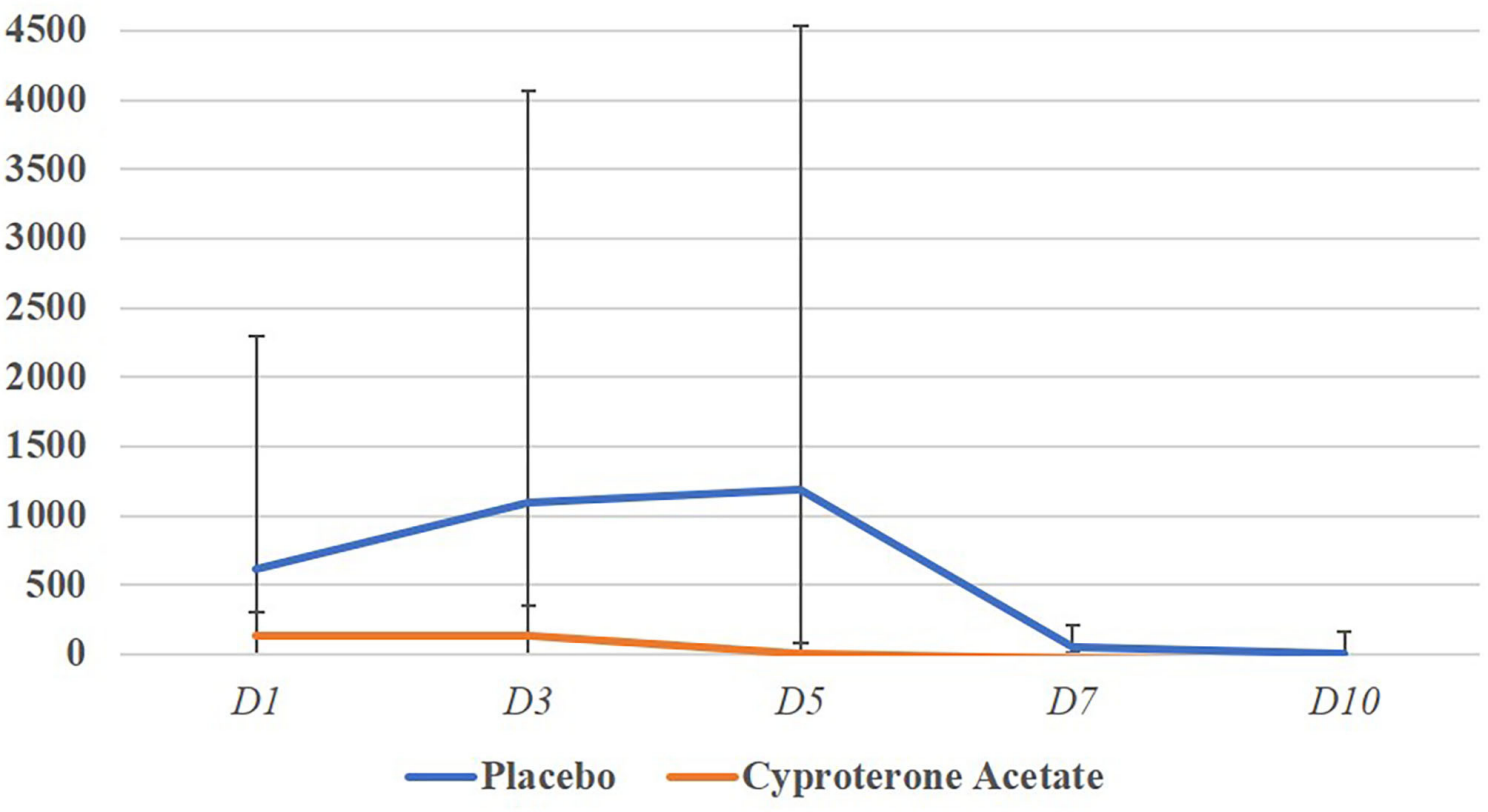
Mean percentage increase in testosterone levels per days and treatment groups.

The subjects' percentage increase in testosterone levels *for the entire duration* of the subjects PFU in the placebo group (*M* = 627.4; *SD* = 1647.2) and the cyproterone acetate group (*M* = 151.8; *SD* = 142.6) were compared using a non-parametric test for independent samples, since Shapiro-Wilk normality tests showed that the data were normally distributed in the cyproterone acetate group (*W* = 0.86; *p* = 0.17) but not in the placebo one (*W* = 0.43; *p* < 0.001). This Mann-Whitney test shows that the percentage increase in testosterone levels on the whole period of the subjects' PFU were not significantly different between the two treatment groups [*U*_(7,9)_ = 38; *p* = 0.54] as shown in Figure 1. Overall, these results suggest that the treatment (placebo or cyproterone acetate) had no significant effect on the start day, end day, duration or amplitude of the PFU.

#### Effects of the Treatment on the BFU

Freeman-Halton extensions of the Fisher exact probability test for a two-rows by two-columns contingency table (see Table 7 above) were performed to examine the relation between the subjects' experience of a BFU (Yes/No) and treatment group (placebo/cyproterone acetate) according to their behavioral scores on the one hand and owners' impression on the other hand, and these two tests showed a significant association between these two variables according both to the veterinarians (*p* = 0.01) and the owners (*p* = 0.03). Indeed, none of the subjects of the cyproterone acetate group against 67% of the subjects of the placebo group experienced a BFU, and these results suggest that cyproterone acetate prevents BFU to occur.

**Table 7:** Numbers of subjects experiencing or not BFU in the two treatment groups according to the veterinarians' scoring (first) and the owners' impression (second).

	**Treatment group**	
**BFU**	**Placebo**	**Cyproterone acetate**
No	3/4	9/9
Yes	6/5	0/0

Because all the subjects experiencing a BFU belonged to the Placebo group, no analyse on the start day, end day, duration, and amplitude of the BFU by treatment group was carried out. However, it should be noted that, in the Placebo group, all but two of the subjects' behavioral scores became at least equal to 0 (the score of reference, 0, attributed at D0) before D10 (*N* = 8, *M* = 2.3, *SD* = 2.4) so that, with two possible exceptions, BFU duration never exceeded a week.

**Figure 2:**
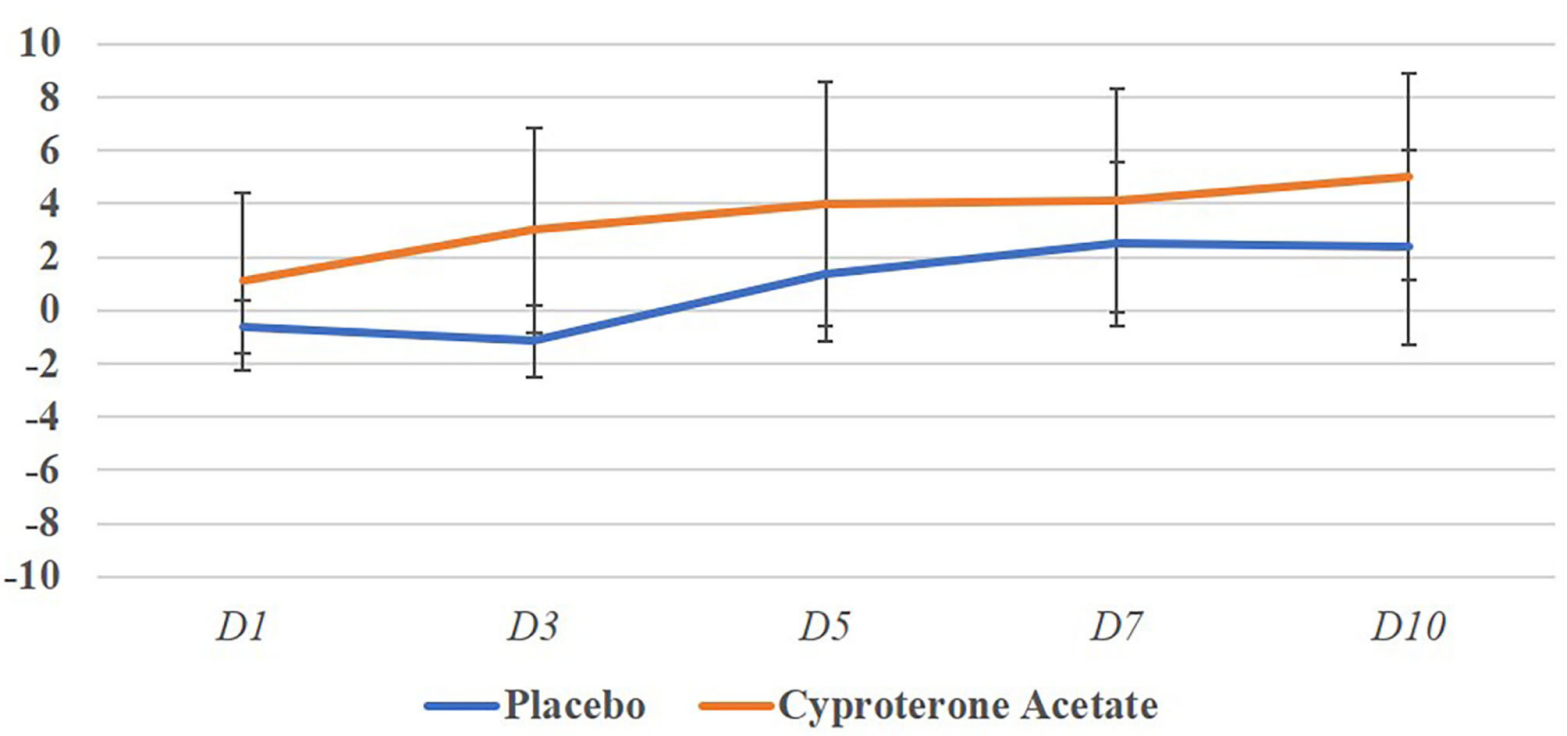
Mean behavioral scores per days and treatment groups.

Figure 2 represents the evolution of the mean behavioral scores per days and treatment groups and shows that despite a great variability can be seen between individuals, the behavioral scores only become negative, i.e., worsening of the behavior in the placebo group.

#### Effects of the Treatment on Behavioral Disorders

As explained above, since none of the subjects of the cyproterone acetate group experienced a BFU, the effects of the treatments on the *deterioration* (evidenced by behavioral scores *inferior* to 0) and, if so, *return to “normalcy”* (evidenced by behavioral scores *equal* to 0) of the subjects' behavior could not be analyzed. Two dogs from the placebo group had a negative score, indicating a *worsening* at 10 days after implantation. However, only one subject in the placebo group and two subjects in the cyproterone acetate group obtained an average behavioral score of 0 (i.e., equal to that of reference, attributed at D0) the last day behavioral scores were measured (i.e., at D10), which incidentally suggests that the behavioral issues of most of the subjects at least partially responded to deslorelin implant under 10 days after implantation Therefore, it seemed of interest to analyze the effects of the treatments on the *improvement* of the subjects' behavior (evidenced by behavioral scores superior to 0) in the 13 subjects with an improved average behavioral scores when compared to their baseline (D0) behavioral score (see Table 8 below).

**Table 8:** Numbers of subjects whose behavioral score was worse (<0), identical (=0) or better (>0) at D10 than that of reference (at D0).

	**Treatment group**	
**Average Behavioral Score at D10**	**Placebo**	**Cyproterone acetate**
<0	2	0
=0	1	2
>0	6	7

First, the subjects' average behavioral scores at D10 (when positive) in the placebo (*N* = 6; *M* = 4.1; *SD* = 3.2) and the cyproterone acetate (*N* = 7; *M* = 6.5; *SD* = 3.0) groups were compared using parametric tests for independent samples since they had normal distributions (Shapiro-Wilk normality tests: *W* = 0.86 and *p* = 0.22 for the placebo group; *W* = 0.80 and *p* = 0.05 for the cyproterone acetate group) and equal variances [*F*-test: *F*_(5,6)_ = 1.08; *p* = 0.45]. This *t*-test showed that the subjects' average behavioral scores were not significantly different between the two treatment groups [*t*_(11)_ = −1.4; *p* = 0.19].

Second, the subjects' days of improvement (i.e., of first positive average behavioral score) in the placebo (*N* = 6; *M* = 5.7; *SD* = 1.0) and the cyproterone acetate (*N* = 7; *M* = 3.3; *SD* = 1.4) groups were compared using non-parametric tests for independent samples since, as shown by Shapiro-Wilk normality tests, they were normally distributed in the cyproterone acetate group (*W* = 0.84 and *p* = 0.12) but were not in the placebo (*W* = 0.64 and *p* < 0.01) group. This Mann-Whitney test shows that the subjects' improvement occurred significantly earlier in the cyproterone acetate group than in the placebo group [*U*_(6,7)_ = 38; approximate *p* = 0.02].

**Figure 3:**
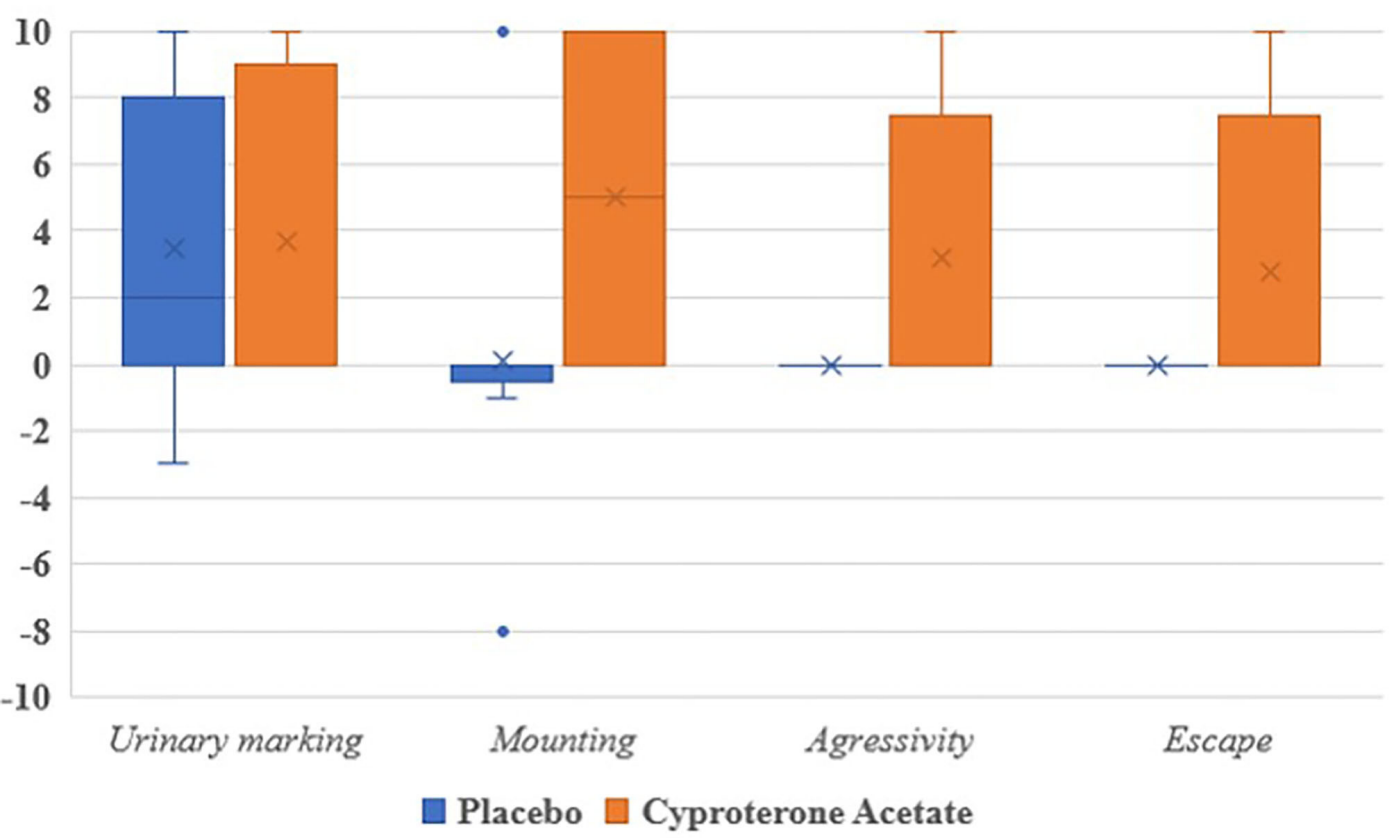
Behavioral scores per symptoms and treatment groups at D10.

Third, because of the very small number of subjects experiencing each of the studied symptoms, no analysis of the subjects' symptomatic behavioral scores (when positive) at D10 was carried out, even though all of them were numerically higher in the cyproterone acetate group than in the placebo group (see Figure 3).

### Secondary Analyses

#### Adverse Effects of the Treatments

No adverse effects were found in any of the subjects of the study, including placebo subjects, nor reported by their owners besides a BFU. Concerning the blood analyses, all samples remained in the normal range. Hepatic values, which were the most susceptible to increase did not change significatively overtime.

#### Dogs' Weight Increases

The Placebo group subjects' weights at D0 (*M* = 21.7; *SD* = 16.0) were compared to both these subjects' weights at D10 (*M* = 21.5; *SD* = 15.8) and these subjects' weights at D28 (*M* = 21.5; *SD* = 15.7) using parametric tests for correlated samples since all data had normal distributions (Shapiro-Wilk normality tests: *W* = 0.90 and *p* = 0.24 at D0; *W* = 0.89 and *p* = 0.19 at D10; *W* = 0.89 and *p* = 0.22). These *t*-tests showed that the Placebo group subjects' weights at D0 were significantly different from neither these subjects' weights at D10 [*t*_(8)_ = 1.25; *p* = 0.25] nor these subjects' weights at D28 [*t*_(8)_ = 0.39; *p* = 0.71].

The cyproterone acetate group subjects' weights at D0 (*M* = 23.4; *SD* = 13.7) were compared to both these subjects' weights at D10 (*M* = 23.8; *SD* = 14.4) and these subjects' weights at D28 (*M* = 23.6; *SD* = 13.6) using parametric tests for correlated samples since all data had normal distributions (Shapiro-Wilk normality tests: *W* = 0.96 and *p* = 0.84 at D0; *W* = 0.97 and *p* = 0.89 at D10; *W* = 0.95 and *p* = 0.72 at D28). These *t*-tests showed that the cyproterone acetate group subjects' weights at D0 were significantly different from neither these subjects' weights at D10 [*t*_(8)_ = −1.24; *p* = 0.25] nor these subjects' weights at D28 [*t*_(8)_ = −0.75; *p* = 0.47].

#### Owners' Satisfaction Scores

The owners' satisfaction scores at D28 in the Placebo group (*M* = 6.3; *SD* = 2.5) and cyproterone acetate group (*M* = 6.3; *SD* = 3.4) were compared using a parametric test for independent samples since they had normal distributions (Shapiro-Wilk normality tests: *W* = 0.89 and *p* =0.19 for the placebo group; *W* = 0.87 and *p* = 0.12 for the cyproterone acetate group) and equal variances [*F*-test: *F*_(8,8)_ = 1.80; *p* = 0.42]. This two-samples *t*-test shows that they were not significantly different between the two treatment groups [*t*_(16)_ = 0; *p* = 1].

Moreover, 67% (6) of the owners in each treatment group were satisfied (with a 7/10 or more evaluation considered as a positive evaluation).

## Discussion

### Cyproterone Acetate Effects on Total Testosterone Levels

This study was conducted assuming that there was a clear link between the BFU and the PFU. Indeed, until the end of the blood testosterone analyses, it seemed correct to assume that cyproterone acetate was efficient to block the PFU, since the cyproterone acetate group had no BFU reported. However, once the testosterone levels were analyzed, this hypothesis had to be rejected and the analyses were limited by two points, not changeable after data collection:

Firstly, the testosterone dosage was total testosterone when the bioactive testosterone only represents the free testosterone. It is therefore possible that this free testosterone level could be different between both groups, but this should be further investigated as it unfortunately could not be measured in this experimental setting.

Secondly, the subjects were not recruited from a behavioral perspective. The criteria for recruiting were (i) owners asking for a chemical castration, (ii) dogs exhibiting at least one of the behavioral symptoms (mounting, marking or aggressivity to other dogs) (iii) dogs aged of 6 months or more so they could be concerned by hormonal puberty. Consequently, in the recruited dogs, some had a good profile for the veterinarian to suspect an androgenic effect on the behavior. For example, a dog that never was aggressive to other dogs and suddenly started to growl when puberty arises would be a good candidate to suspect a testosterone effect, hence a possible improvement when implanted with deslorelin. Conversely, a dog that has always been exhibiting fearful behavior to other dogs and became more and more aggressive toward all dogs over time, would not be expected to change this behavior if implanted with deslorelin. However, owners still could ask for this implant because castration is often seen as a possible solution to reduce aggressivity and impulsivity in dogs by their owners. In this perspective, there is a major need of field studies investigating the behavioral profiles of the dogs before deslorelin implantation, to help owners and veterinarians to determinate whether the castration might solve their behavioral issue or not. It has to be noted that improvement of the dog's behavior was not expected by the veterinarian for all included dogs. Consequently, the efficacy of the cyproterone acetate treatment or deslorelin implant on improving undesired behaviors could not be analyzed because it would have needed a much narrower targeting of the subjects beforehand.

This latter point underlines the interest of using deslorelin implant instead of surgical castration, because it is a good test to assess the behavioral effects. In the cases where no behavioral effects were observed, the chemical effects of castration would wear off after 6 months and the dog can stay entire. However, the percentage of owners considering that the treatment solved their dog's behavioral issue happened to be high in both groups (67%), showing that deslorelin was indeed efficient in over half of the cases.

Because our hypothesis of a link between total testosterone level and BFU could not be demonstrated in our experimental setting, we had to design two different scores (i.e., PFU and BFU) to be able to separately analyse these two phenomena that were not linked as we expected them to be. Moreover, our results showed that they do not co-occur and that the total testosterone level measured was not necessarily a good indicator, or at least not the only one to characterize the FU effect. One explanation could be that the FU effect is due to a testosterone metabolite rather than total testosterone, which could not be effectively measured in our experimental set up.

On the contrary, our results showed that cyproterone acetate had no effect on the physiological parameter of total testosterone in the blood. Cyproterone acetate's peripherical mechanism of action consists in blocking testosterone receptors in the cellular cytosol with cyproterone acetate acting as a competitive inhibitor of testosterone. This action does not affect the level of circulating testosterone. However, cyproterone acetate also acts in the brain by blocking the secretion of GnRH and therefore secondarily that of FSH, LH, and testosterone. This second effect should be observed. This may not be the case because the action of cyproterone acetate is counterbalanced by that of deslorelin (i.e., increased FSH and LH during the FU effect). Nevertheless, a behavioral effect was observed in our study, which suggests that the BFU effect did not solely and/or fully depend on total testosterone. Several mechanisms of action of cyproterone acetate have been reported to directly interact with GABA, serotonin and dopamine. It is possible that the observed behavioral effects in our study could be the result of such mechanisms of action, rather than the peripherical ones and this would explain why testosterone levels were not altered in this study.

Hence, further experiments are required to fully understand the FU effect's mechanisms: using cyproterone acetate alone would allow to test if the behavioral control of the behavior would happen without the desloreline, but this option is not realistic considering the long term effects of cyproterone acetate. Another more realistic and safer option thus would be to use other neuroleptics, such as risperidone, to see if the central psychotropic effect is sufficient to control the BFU.

### Cyproterone Acetate Effects on Behavioral Flare-Up

However, our results demonstrated that cyproterone acetate administered for 8 days after the deslorelin implantation at a dose of 2 mg/kg bid was a good solution to remove the BFU. Indeed, none of the dogs receiving the cyproterone acetate treatment experienced a BFU effect, whereas 56% (according to the veterinarian) or 66% (according to owners) experienced a visible BFU in the placebo group.

Our results showed no quantitative difference on the behavioral improvement between cyproterone acetate and placebo, but the subjects' improvement occurred significantly earlier in the cyproterone acetate group than in the placebo group. In other words, our results suggested that cyproterone acetate did not only prevent the BFU but also fasten the onset point of the positive effects of deslorelin implant, i.e., removing some undesired behaviors. Unfortunately, due to the limited sample size, additional studies would be required for each monitored symptom of this study.

One point to take into consideration for future research is the difficulty to precisely and quantitatively assess certain behaviors compared to others. Urine marking for example is very easy to monitor, whereas aggression toward other dogs is much more difficult to measure experimentally (owners might avoid this behavior by avoiding other dogs every day). Also, as stated before, further studies on behavior should try and discriminate between the dogs in which a castration effect is expected from those where in which it does not.

### Safety of Cyproterone Acetate

Cyproterone acetate was reported to present side effects, especially on the liver in long-term use (43, 44). During this study, side effects were closely monitored at each visit and blood samples were collected. No side effects were reported and no change in the analyses was recorded either, which supported the safe use of cyproterone acetate on such a short-term use. Moreover, pharmacovigilance files were filled up for six dogs that encountered a worsening of their symptoms, but all these effects happened in the placebo group and thus cannot be attributed to cyproterone acetate use. However, our sample was not large enough to guarantee a perfect safety and the use of cyproterone acetate in this indication on a larger sample of dogs would be required to confirm these first results.

Conversely, several side effects were noted in the placebo group. They all belonged to the expected FU effects category, such as an increase in the occurrence of mounting, urine marking, aggressivity to other dogs or escaping. One dog in the placebo group even had an apparition of mounting behavior.

In a clinical perspective, in case of veterinary use of cyproterone acetate to prevent a possible BFU on a dog that is about to receive deslorelin implantation, it would appear necessary to inform the owners about the possible long-term effects of cyproterone acetate and the final choice of using cyproterone acetate should be made with a specific benefice/risk assessment. Indeed, many FU effects, such as aggressivity toward other dogs, can put the dog's health at risk and the use of cyproterone acetate for a few days might be less risky than not using it.

There was no significant weight gain during the time course of the study. Similarly, the weight's gain usually reported with orchiectomy or chemical castration could have been more important in the cyproterone acetate, but it was not. The agonistic effect of deslorelin in the FU phase could explain why there is no weight gain during the study. However, it would have been interesting to evaluate whether the appetite change, rather than just weight, was different in the two groups.

There is currently an increasing interest around medical castration for several reasons: it is reversible, it is a safe way to test the possible effects of an orchiectomy without taking the risks of anesthesia and is ethically more acceptable as there is not any organ removal from the animal. Hence, there is a need for more experimental studies systematically exploring the difference of effects between medical and surgical castration. This should be done by assessing all the types of effects: on possible weight gain, on the management of sex organ related diseases and on behavior, along with evaluating any risk of side-effects. This alone is not always easy to achieve, because some effects can only be observed very late in the animal life and would require following a very large number of subjects during their lifetime. Another way to further investigate this would be to monitor the same dog implanted then castrated, keeping in mind the unavoidable bias that deslorelin-related effects from implantation might change the orchiectomy effects.

## Conclusion

Our study suggested that cyproterone acetate administered during a week at 2 mg/kg bid was effective and safe to supress the BFU. Surprisingly, these effects were not correlated with the total testosterone concentrations. Cyproterone acetate effects might be due to its central action of on serotonin and other neuromotransmitters, rather than its peripherical effect on testosterone, therefore suggesting that testosterone should rather be seen as a behavioral modulator rather than a behavioral inducer.

## Data Availability Statement

The original contributions presented in the study are included in the article/Supplementary Material, further inquiries can be directed to the corresponding author/s.

## Ethics Statement

The animal study was reviewed and approved by an independent yet internal Ethical Review Committee (ERC# 2OL9O9-1), stating that the protocol complied with the European Directive # 2010-63-EU, the application of the 3Rs principles and the internal Code of Animal Care, as the study occurred in privately owned veterinarian clinics. Written informed consent was obtained from the owners for the participation of their animals in this study.

## Author Contributions

The idea for the paper was conceived by SM, XL, CF, and ER. The experiments were designed by SM, XL, TM, and CF. The experiments were performed by SM and XL. The data were analyzed by SM and TM. The paper was written and reviewed by SM, TM, CF, ER, and XL. All authors contributed to the article and approved the submitted version.

## Funding

The trial reported in this article was partially financially supported by Virbac, manufacturer of Suprelorin. This sponsor had however no influence on the results of this trial.

## Conflict of Interest

ER and CF were employees of Virbac, manufacturer of Suprelorin, at the time of the study. The remaining authors declare that the research was conducted in the absence of any commercial or financial relationships that could be construed as a potential conflict of interest.

## Publisher's Note

All claims expressed in this article are solely those of the authors and do not necessarily represent those of their affiliated organizations, or those of the publisher, the editors and the reviewers. Any product that may be evaluated in this article, or claim that may be made by its manufacturer, is not guaranteed or endorsed by the publisher.
