# Supplementary material for: Double-Blind, Placebo-Controlled Trial of Cyproterone Acetate to Prevent Flare-Up Effect on Dogs Implanted With Deslorelin
Source: Front Vet Sci. 2021 Sep 29;8:714154. doi: 10.3389/fvets.2021.714154 (PMC8511793; doi:10.3389/fvets.2021.714154)
Supplement: Supplementary file 1 [file Presentation_1.PDF]

VETERINARY PHARMACOVIGILANCE
FORM FOR REPORTING SUSPECTED ADVERSE EVENTS

1 - SUBSIDIARY OR COMPANY:	2 - ORIGINAL REPORTER
Name of sender :<br>**Country :**<br>Case reference :<br>Type of report : ☐ Initial ☐ Follow-up<br>**Date of First info Receipt (DFR) :**	Name: Firstname:<br>Address:<br>Telephone / Fax / Email :<br>**Occur country :**<br>☐ Veterinarian ☐ Physician ☐ Pharmacist ☐ Owner<br>☐ Other :

If the original reporter does not agree that his/her complete name and address are sent to MAH, please tick this box ☐

3 - ☐ VETERINARIAN ☐ PHARMACIST ☐ PHYSICIAN	4 - ☐ ANIMAL OWNER ☐ HUMAN PATIENT
Name : Firstname:<br>Address :<br>Telephone/ Email:<br>☐ Identical to original reporter	Name : Firstname:<br>Address :<br>Telephone/ Email:<br>☐ Identical to original reporter

5 - ANIMAL DATA

N° of animals treated/exposed : **N° of animals affected :** **N° of animals dead :**

Affected animals characteristics : Species: Identification :

Breed/production type : Weight : Age :

Sex/physiological status : ☐ Female ☐ Male ☐ Unknown / ☐ Pregnant ☐ Neutered ☐ Lactating ☐ Unknown

State of health at time of treatment : ☐ Good ☐ Fair ☐ Poor ☐ Critical ☐ Unknown

Concomitant medical conditions:

6 - PRODUCTS DATA AND TREATMENT DETAILS

List of all relevant medication(s) administered before the event (one product per column; if more products are concerned, please use extra sheet) – *NA = Not applicable – Unk = Unknown*

**Product name<br>Company name<br>MA number**				
**Pharmaceutical form & concentration**				
**Batch Number<br>Expiry date**				
**Stored correctly?<br>If No, explain**	☐Yes ☐No ☐ Unk	☐Yes ☐No ☐ Unk	☐Yes ☐No ☐ Unk	☐Yes ☐No ☐ Unk
**Dose & Frequency of the administered treatment**				
**Used Route & Administration site**				
**Treatment administered by (veterinarian, owner, …)**				
**Reason for use or initial diagnosis?**				
**Use according to label ?<br>if No, explain**	☐Yes ☐No ☐ Unk	☐Yes ☐No ☐ Unk	☐Yes ☐No ☐ Unk	☐Yes ☐No ☐ Unk
**Start date of treatment**				
**Stop date or duration of treatment**				
**Action after event (drug withdrawn, dose reduced)?**				
**Did event abate after stopping drug treatment?**	☐Yes ☐No<br>☐NA ☐Unk	☐Yes ☐No<br>☐NA ☐Unk	☐Yes ☐No<br>☐NA ☐Unk	☐Yes ☐No<br>☐NA ☐Unk
**Did event reappear after reintroduction?**	☐Yes ☐No<br>☐NA ☐Unk	☐Yes ☐No<br>☐NA ☐Unk	☐Yes ☐No<br>☐NA ☐Unk	☐Yes ☐No<br>☐NA ☐Unk
**Is the event related to this product according to original reporter?**	☐Yes ☐No ☐Unk	☐Yes ☐No ☐Unk	☐Yes ☐No ☐Unk	☐Yes ☐No ☐Unk

DOC APPLICABLE

7 - <u>EVENT DATA</u>

☐ **Safety issue in animals** ☐ **Lack of expected efficacy** ☐ **Withdrawal period issue**
☐ **Environmental problem** ☐ **Transmission of infectious agents**

**CHRONOLOGY**		
**Date of onset of event :**	**Time to onset between the start of exposition and the event (in seconds, minutes, days,…):**	**Duration of the event (in seconds, minutes, days,…):**

Describe the sequence of events incl. administration of product(s), all clinical signs, site of reaction, severity, laboratory tests, necropsy results, possible contributing factors (if necessary use extra sheet) :

Treatment given to address this adverse event : ☐ Yes ☐ No Details :

Outcome of event to date :

	Euthanized	Died	On going reaction	Under treatment	Recovered without sequelae	Recovered with sequelae	Unknown
N◦ of animals :							☐
Date when :							☐

Has reporter seen similar adverse events before with other product(s) on this (these) animal(s) ? ☐Yes ☐No ☐unknown (if yes, describe):

8 - <u>PREVIOUS EXPOSURE AND EVENT(S) TO PRODUCT(S) :</u>

Previous exposure to product(s) ? ☐ Yes ☐ No Which one(s): Date :

Previous reaction to product(s) ? ☐ Yes ☐ No Which one(s): Date :

Description of event, treatment given and outcome :

9 - <u>DETAILS OF SUSPECTED ADVERSE EVENT(S) IN HUMANS</u>

Sex : Age/Date of birth : Occupation (with relevance to exposure) :

Physiological status : ☐ Pregnant ☐ Breastfeeding ☐ Unknown

Date of exposure : Date of reaction :

Nature and duration of exposure, reaction details (including symptoms and treatment of the reaction) and outcome :

Identification of the physician or poison center or pharmacovigilance center if consulted :

10 - <u>FOR SUBSIDIARY OR COMPANY USE / CAUSALITY ASSESSEMENT</u>

☐ **A** (probable) ☐ **B** (possible) ☐ **O** (unclassified) ☐ **O1** (inconclusive) ☐ **N** (unlikely)
Reasons for assessment and comments :

Name of the original reporter (see section 2 of the document) or the person responsible for completing this form :

Date : **Signature (if reporting form printed) :**

☐ Attachments included *☐ Reports to follow :*

Has competent authority or pharmacovigilance center been notified with this case? ☐ Yes ☐ No ☐ Unknown

DOC APPLICABLE
