# Supplementary material for: Double-Blind, Placebo-Controlled Trial of Cyproterone Acetate to Prevent Flare-Up Effect on Dogs Implanted With Deslorelin
Source: Front Vet Sci. 2021 Sep 29;8:714154. doi: 10.3389/fvets.2021.714154 (PMC8511793; doi:10.3389/fvets.2021.714154)
Supplement: Supplementary file 2 [file Presentation_2.PDF]

Owner informed consent

Dog's name: ____________________

Project Title: Double-blind, placebo-controlled trial of cyproterone acetate to prevent flare-up effect on dogs implanted with deslorelin

Appointments: I will come to the veterinary clinic six times (day 1, day 3, day 5, day 7, day 10, day 28), starting today (day 0). I have been informed of the examinations that will be carried out on my dog: behavior score (day 0, day 1, day 3, day 5, day 7, day 10, day 28), and blood samples (day 0, day 1, day 3, day 5, day 7, day 10). Two telephone interviews (day 14 and day 21) are scheduled to record my dog's behavioral score.

Risks associated with the study: side effects associated with taking the medication and placing the implant (anorexia, increased appetite, lethargy, excitement, decreased motivation, increased motivation)

Expected Duration of Participation and Withdrawal from Participation: Your participation in this study is completely voluntary and you may withdraw your dog from the study at any time without jeopardizing your relationship with your veterinarian. If you no longer wish to participate, you do not have to provide a reason for your decision, and you will not lose any benefit regarding the veterinary care given to your dog. If you remove your animal from the research project, all samples taken during the study may be kept for analysis. Your animal's blood samples will be identified by their name and unique number. You agree that the Virbac laboratory owns all rights to the samples, and you waive any right of inspection or approval with regard to their use. Your personal details will only be available from your veterinarian and at no time will the Virbac laboratory have access to this information. All contact between you and the Virbac laboratory will be made by your veterinarian. If this study is published, only a summary of the results will be visible and your identity or that of your dog will not be revealed.

Confidentiality: the information collected during this study will be confidential and will not be used for any other purpose. These results could be published in a scientific journal.

Acceptance: in light of the information presented in this document, I accept that my dog ________________________ will participate in this study

Date: _____________________

Signatures:

Main researcher

Owner's name

Feuille de suivi

(To give to the owner)

Dog's name: ____________________

Stamp of the clinic:

Phone number:

E-mail of your veterinary:

Please don't hesitate to ask if you have any question,

We thank you for your participation into this clinical study!

Follow-up dates

	DATE	Veterinary visit	Blood sample	Phone call
Day 0		X	X	
Day 1		X	X	
Day 3		X	X	
Day 5		X	X	
Day 7		X	X	
Day 10		X	X	
Day 14				X
Day 21				X
Day 28		X		
